# History and Familial Aggregation of Immune-Mediated Diseases in Sarcoidosis: A Register-Based Case-Control-Family Study

**DOI:** 10.1016/j.chest.2024.05.014

**Published:** 2024-07-08

**Authors:** Marios Rossides, Susanna Kullberg, Elizabeth V. Arkema

**Affiliations:** a Unit of Epidemiology, Institute of Environmental Medicine, Karolinska Institutet, Stockholm, Sweden; b Division of Respiratory Medicine, Department of Medicine Solna, Karolinska Institutet, Stockholm, Sweden; c Clinical Epidemiology Division, Department of Medicine Solna, Karolinska Institutet, Stockholm, Sweden; d Department of Respiratory Medicine and Allergy, Theme Inflammation and Ageing, Karolinska University Hospital, Stockholm, Sweden

**Keywords:** autoimmune diseases, case-control studies, heritability, sarcoidosis, Sweden

## Abstract

**Background:**

An autoimmune component in the cause of sarcoidosis has long been debated, but population-based data on the clustering of immune-mediated diseases (IMDs) and sarcoidosis in individuals and families suggestive of shared cause are limited.

**Research Question:**

Do patients with a history of IMDs have a higher risk of sarcoidosis and do IMDs cluster in families with sarcoidosis?

**Study Design and Methods:**

We conducted a case-control-family study (2001-2020). Patients with sarcoidosis (N = 14,146) were identified in the Swedish National Patient Register using a previously validated definition (≥ 2 International Classification of Diseases [ICD]-coded inpatient or outpatient visits). At diagnosis, patients were matched to up to 10 control participants from the general population (N = 118,478) for birth year, sex, and residential location. Patients, control participants, and their first-degree relatives (FDRs; Multi-Generation Register) were ascertained for IMDs by means of ICD codes in the Patient Register (1968-2020). Conditional logistic regression was used to estimate ORs and 95% CIs of sarcoidosis associated with a history of IMDs in patients and control participants and in FDRs.

**Results:**

Patients with sarcoidosis exhibited a higher prevalence of IMDs compared with control participants (7.7% vs 4.7%), especially connective tissue diseases, cytopenia, and celiac disease. Familial aggregation was observed across IMDs; the strongest association was with celiac disease (OR, 2.09; 95% CI, 1.22-3.58), followed by cytopenia (OR, 1.88; 95% CI, 0.97-3.65), thyroiditis (OR, 1.72; 95% CI, 1.14-2.60), skin psoriasis (OR, 1.70; 95% CI, 1.34-2.15), inflammatory bowel disease (OR, 1.53; 95% CI, 1.14-2.03), immune-mediated arthritis (OR, 1.49; 95% CI, 1.20-1.85), and connective tissue disease (OR, 1.39; 95% CI, 1.00-1.93).

**Interpretation:**

This study showed that IMDs confer a higher risk of sarcoidosis and they aggregate in families with sarcoidosis, signaling a shared cause between IMDs and sarcoidosis. Our findings warrant further evaluation of shared genetic mechanisms.

**Take-home Points**

**Study Question:** Do immune-mediated diseases (IMDs) in patients and their relatives pose a higher risk of sarcoidosis diagnosis and what are the implications for sarcoidosis cause?

**Results:** The results of this study showed that IMDs are more prevalent in patients with sarcoidosis at the time of diagnosis compared with individuals from the general population of similar age and sex. IMDs cluster in families with sarcoidosis; having a first-degree relative with an IMD increases the likelihood of developing sarcoidosis by 50% to 100%.

**Interpretation:** We found that sarcoidosis coaggregates with IMDs, indicating a shared cause between IMDs and sarcoidosis. These findings warrant investigation of shared genetic mechanisms.

Sarcoidosis, a systemic disease of unknown cause, is believed to occur in genetically predisposed individuals in whom one or several antigens trigger a sustained granulomatous inflammation that leads to clinical disease.^1^ Sarcoidosis shares many clinical and pathophysiologic features of immune-mediated diseases (IMDs), such as involvement of genes related to the immune system, considerable variation in epidemiologic features, clinical presentation, prognosis, and treatment approach. Of note, no antigen or autoantibodies have been identified so far, but this is now a matter of intense research.^2^

Several studies, which recently were reviewed extensively by Arkema et al,^3^ observed a higher prevalence of IMDs in individuals with sarcoidosis compared with the general population.^4^, ^5^, ^6^, ^7^ In some, up to 12% of individuals with sarcoidosis received a diagnosis of another IMD, such as rheumatoid arthritis or systemic lupus erythematosus, or showed features of them, such as disease specific autoantibodies.^4^ It is not entirely clear why individuals with sarcoidosis may demonstrate (other) IMDs. Among other factors, genetic and environmental factors that are shared among the spectrum of IMDs are suspected to explain, at least partly, the clustering of these diseases in individuals.

Coaggregation of sarcoidosis and IMDs in families would suggested shared genetic and environmental pathways among these diseases.^3^ Indeed, a series of register-based Swedish studies suggested familial clustering of sarcoidosis and several IMDs, such as diabetes mellitus type 1,^8^ Graves disease,^9^ rheumatoid arthritis,^10^ and inflammatory bowel diseases,^11^ and multiple sclerosis.^12^ In these studies, data on disease were limited to hospitalizations and no control group was recruited for comparison purposes. Using similar data from Sweden, first-degree relatives (FDRs) of patients with celiac disease were 1.6-fold more likely to develop sarcoidosis than FDRs of individuals without celiac disease.^13^

Identifying whether and which IMDs cluster in individuals and families of individuals with sarcoidosis reveals similarities and differences among those diseases and is a valuable way to guide further research. Such observations may improve clinical practice by increasing awareness among treating physicians and potentially may improve diagnosis and treatment. Therefore, we conducted a large, population-based study with two objectives: (1) to identify whether a history of IMDs is associated with higher odds of sarcoidosis and (2) to explore and quantify the familial clustering of IMDs in sarcoidosis by estimating ORs of sarcoidosis associated with having FDRs with diagnoses of IMDs.

## Study Design and Methods

We conducted a case-control family study using nationwide Swedish register data.

### Patients and General Population Control Participants

Sarcoidosis patients were identified from the National Patient Register (NPR) using International Classification of Diseases (ICD)-coded hospitalizations and outpatient visits at hospitals (excluding primary care visits). The NPR component collecting data on hospitalizations was established in 1968 and gained nationwide coverage in 1987, whereas outpatient non-primary care visits for somatic diseases have been registered on a nationwide level since 2001.^14^ We required at least two visits listing an ICD, 10th Revision, code for sarcoidosis (D86) between 2001 and 2020. Patients with a first visit before 2001 were excluded to capture patients with newly diagnosed disease. This sarcoidosis definition recently was found to be highly valid (positive predictive value, 94%).^15^ The second visit for sarcoidosis was deemed the index date.

In the registers, we lack information on sarcoidosis phenotype, stage, and disease severity. Treatment with systemic corticosteroids and steroid-sparing methotrexate or azathioprine mainly is reserved for patients with severe phenotypes.^1^^,^^16^ We used dispensation of at least one of these treatments around the time of diagnosis (± 3 months from the first visit for sarcoidosis) as a marker of sarcoidosis severity around diagnosis. Data on medication dispensations were obtained from the National Prescribed Drug Register using Anatomical Therapeutic Chemical codes (H02AB01/02/04/06/07, L01BA01, L04AX03, and L04AX01) and were available starting July 2005. Thus, only patients who received a diagnosis from 2006 and onward could be classified as treated or not treated around the time of diagnosis.

At the index date, patients with sarcoidosis were matched individually to up to 10 general population control participants without a history of sarcoidosis in the NPR on birth year, sex, and residential location. Control participants were identified using the Total Population Register and had to be living in Sweden at index date. Patients and control participants are collectively referred to as *probands*, that is, index individuals whose relatives were identified and ascertained for IMDs.

To reduce possible misdiagnosis of sarcoidosis further, we excluded patients and control participants younger than 18 years or older than 85 years at the index date and those with a hematopoietic or lung cancer diagnosis in the National Cancer Register within 6 months before or after the first visit for sarcoidosis or the corresponding date in control participants (ICD, Seventh Revision, codes 162, 163, and 200-205). We further excluded probands born outside Sweden, aiming to restrict to a genetically more homogenous population and to ensure that probands’ relatives could be identified in registers.

### First-Degree Relatives

We identified probands’ FDRs, that is, biological parents, full siblings, or biological offspring, using the Multi-Generation Register. The register links individuals born after 1932 and alive in 1961 or later to their biological parents.^17^ The number of FDRs (overall and by type) on average was similar between patients and control participants, which warranted that potential familial aggregation of diseases was not influenced by dissimilarities in family size. All analyses were restricted to probands who had at least one FDR, because probands without relatives do not by definition contribute any useful information on familial risks.

We received ethical permission for this study from the Swedish Ethical Review Authority (protocol no. 2020-00437), which waived the need to collect informed consent from participating individuals.

### Immune-Mediated Diseases

We ascertained FDRs for IMDs using ICD-coded data on hospitalizations and outpatient visits from the NPR (Table 1). We required at least two inpatient or outpatient visits between 1968 and 2020, and we considered all diagnoses regardless of whether they occurred before or after the diagnosis of sarcoidosis or matching in the proband. To maximize estimation power because some of these diseases are rare, we further grouped them into immune-mediated arthritis (any of rheumatoid arthritis, juvenile idiopathic arthritis, ankylosing spondylitis, or psoriatic arthritis), connective tissue diseases (any of systemic lupus erythematosus, Sjögrens syndrome, systemic sclerosis, immune myopathies, or vasculitis), immune-mediated thyroiditis (autoimmune thyroiditis or Graves disease), immune-mediated CNS disorders and neuropathies (any of Guillain-Barré syndrome, multiple sclerosis, or myasthenia gravis), and immune-mediated cytopenia (including immune hemolytic and pernicious anemia and immune thrombocytopenic purpura). An FDR could have been diagnosed with several diseases and therefore contribute several times in the analysis for each IMD. We also evaluated the history of IMDs in probands defined as at least two visits in the NPR before the first visit for sarcoidosis or corresponding date for control participants.

**Table 1:** IMDs Ascertained in Probands and First-Degree Relatives in the National Patient Register Using ICD Codes

Disease According to Disease Group	ICD Code^a^		
	10th Revision	Ninth Revision	Eighth Revision
Sarcoidosis	D86	135	135
Immune-mediated arthritis			
Rheumatoid arthritis	M05, M06.0, M06.2, M06.3, M06.8, M06.9, M12.3	714A-714C, 714W, 719D	712,10, 712,20, 712,38, 712,39
Juvenile idiopathic arthritis	M08, M09	714D	712,0
Ankylosing spondylitis	M45	720A	712,40, 726,99
Psoriatic arthritis	M07.0, M07.3, L40.5	713D, 696A	696,00
Connective tissue disease			
Systemic lupus erythematosus	M32.1, M32.8	710A	734,10
Sjögrens syndrome	M35.0	710C	716
Systemic sclerosis	M34	710B	734,0
Inflammatory myopathies	M33	710D, 710E	716
Vasculitis	M30, M31	446	446
Inflammatory bowel diseases	K50, K51	555, 556	563,00, 563,10, 569,02
Celiac disease	K90.0	579A	269,10
Immune-mediated thyroiditis			
Immune thyroiditis (Hashimoto’s disease)	E06.3	245C	245,03
Graves disease	E05.0	242A	242,00
Immune-mediated central nervous system disorders or neuropathies			
Guillain-Barré syndrome	G61.0	357A	357
Multiple sclerosis	G35	340	340
Myasthenia gravis	G70.0	358A	733,00
Diabetes mellitus type 1	E40	250	250
Addison disease	E27.0	255E	255,10
Psoriasis	L40	696	696
Immune-mediated cytopenia			
Immune-mediated anemia	D51.0, D59.1	281A, 283A	281,10, 283,90
Immune thrombocytopenic purpura	D69.3	287D, 446G	287,10, 446,40

ICD = International Classification of Diseases; IMD = immune-mediated disease.

a ICD, 10th Revision, codes were used during the calendar years 1997 through 2020; ICD, Ninth Revision, codes were used from 1987 through 1996; and ICD, Eighth Revision, codes were used from 1969 through 1986.

We additionally examined familial aggregation of sarcoidosis to benchmark our findings on familial coaggregation of IMDs. We previously examined the familial aggregation of sarcoidosis in patients who received a diagnosis until 2013 using a slightly different sarcoidosis definition that also included inpatient data from the NPR before 2001,^18^ capturing newly diagnosed and prevalent cases of sarcoidosis, unlike in this study, in which all cases were newly diagnosed.

### Other Variables

We obtained probands and their FDRs’ birth years (to calculate age) and sex from the Total Population Register. From the Education Register, we retrieved probands’ years of completed education during the year before first sarcoidosis visit or the corresponding year in control participants (≤ 9 years, 10-12 years, ≥ 13 years, or missing).

### Statistical Analysis

We estimated ORs and 95% CIs of sarcoidosis in the probands associated with a history of any IMD or disease group (Table 1) in the proband (patient or control participant). Conditional logistic regression models were adjusted inherently for matching variables birth year, sex, and residential location. We also estimated ORs and 95% CIs of sarcoidosis associated with any IMD or IMD disease groups in FDRs (Table 1) using conditional logistic regression models in which each FDR contributed one observation (exposed or unexposed based on disease status). Except for matching variables (age, sex, and residential location), models were not adjusted further. CIs were constructed using robust variance estimators accounting for the family clustering. Analyses were stratified further by kinship (parent, full sibling, or offspring), sex of the proband, and sarcoidosis treatment status around diagnosis (treated or untreated). Modification of ORs by each of the three factors was tested using likelihood ratio tests, with a *P* value of < .05 indicating statistically significant differences. Low numbers did not allow investigation of effect modification for immune-mediated cytopenia.

Analyses were considered exploratory, so estimates were not corrected for multiple testing. Missing data for the variable of education were presented in descriptive analysis; no further analysis or imputation of missing was performed because these variables were not used in statistical modeling. Data were managed and analyzed using SAS version 9.4 software (SAS Institute, Inc.) using, among others, the procedures means, freq, and phreg. Plots were created using the package ggplot2^19^ for R version 4.3.3 software (R Foundation for Statistical Computing).

## Results

We included 14,146 patients with sarcoidosis and 118,478 matched control participants in this analysis (mean age, 51 years; female sex, 42%). We identified on average five FDRs per patient or control participant, including > 700,000 FDRs in total who were ascertained for IMDs. Demographic and clinical characteristics of patients, control participants, and FDRs are presented in Table 2.

**Table 2:** Characteristics of Proband Patients With Sarcoidosis, Their Matched General Population Control Participants, and Patients’ and Control Participants’ FDRs

Variable	Sarcoidosis Probands	Control Participant Probands
Probands		
No.	14,146	118,478
Age at diagnosis or matching, y	50.9 ± 14.8	51.1 ± 14.7
Sex		
Female	5,968 (42.2)	49,768 (42.0)
Male	8,178 (57.8)	68,710 (58.0)
Education, y		
≤ 9	2,731 (19.3)	22,804 (19.2)
10-12	7,161 (50.6)	57,475 (48.5)
≥ 13	4,206 (29.7)	37,874 (32.0)
Missing	17 (0.1)	312 (0.3)
Sarcoidosis treatment around diagnosis^a^	(n = 10,342)	NA
Yes	4,194 (59.5)	NA
No	6,148 (40.6)	NA
FDRs		
No.	73,860	618,106
No. per proband	5.2 ± 2.1	5.2 ± 2.0
Age at proband’s diagnosis or matching, y	51.1 ± 28.6	51.2 ± 28.7
Sex		
Female	36,439 (49.3)	305,249 (49.4)
Male	37,421 (50.7)	312,857 (50.6)
Birth year, median (IQR)	1958 (1939-1980)	1958 (1939-1980)

Data are presented as No. (%) or mean ± SD unless otherwise indicated. Percentages may not sum up to 100 owing to rounding. FDR = first-degree relative; IQR = interquartile range; NA = not applicable.

a Refers to a dispensation of at least one prescribed systemic corticosteroid treatment, methotrexate, or azathioprine ± 3 mos from the time of first visit for sarcoidosis. Data available only for patients who received a diagnosis from 2006 and onward.

### History of IMDs

At sarcoidosis diagnosis, 7.7% of patients had a history of at least one IMD compared with 4.7% of the general population of control participants. As in the general population, the most common IMDs diagnosed before sarcoidosis were diabetes mellitus type 1 (2.3%), immune-mediated arthritis (1.6%), and inflammatory bowel diseases and psoriasis (1.4% each). Table 3 shows ORs of sarcoidosis associated with a history of an IMD. Having a history of any IMD was associated with 79% higher odds of sarcoidosis (OR, 1.79; 95% CI, 1.73-1.85). Specifically, immune-mediated cytopenia was more than four times more common (OR, 4.59; 95% CI, 3.76-5.61) and connective tissue diseases and celiac disease were about three times more prevalent in patients than in control participants (OR, 3.23 [95% CI, 2.91-3.59] and 2.96 [95% CI, 2.54-3.44], respectively). In the connective tissue disease group, ORs of sarcoidosis were threefold higher in individuals with a history of vasculitis (OR, 3.87), immune myopathies (OR, 3.86), Sjögrens syndrome (OR, 3.56), and systemic lupus erythematosus (OR, 2.35). For other IMDs, history of diabetes mellitus type 1 was associated with twofold higher odds of sarcoidosis (OR, 1.97; 95% CI, 1.85-2.09), followed by inflammatory bowel disease (OR, 1.62; 95% CI, 1.50-1.74), immune-mediated arthritis (OR, 1.58; 95% CI, 1.47-1.69), skin psoriasis (OR, 1.54; 95% CI, 1.43-1.66), immune-mediated CNS disorders or neuropathies (OR, 1.39; 95% CI, 1.22-1.59), and immune-mediated thyroiditis (OR, 1.36; 95% CI, 1.18-1.56).

**Table 3:** ORs of Sarcoidosis in Probands Associated With Having a History of IMD in Index Individuals (Probands)

Variable	Probands With Sarcoidosis	Control Probands	OR (95% CI)
No. of probands	14,146	118,478	NA
History of IMD^a^			
Any	1,091 (7.7)	5,579 (4.7)	1.79 (1.73-1.85)
Immune-mediated arthritis	228 (1.6)	1,281 (1.1)	1.58 (1.47-1.69)
Rheumatoid arthritis	118 (0.8)	757 (0.6)	1.38 (1.26-1.52)
Juvenile idiopathic arthritis	14 (0.1)	64 (0.1)	1.58 (1.17-2.13)
Ankylosing spondylitis	41 (0.3)	204 (0.2)	1.95 (1.66-2.29)
Psoriatic arthritis	72 (0.5)	344 (0.3)	1.84 (1.63-2.08)
Connective tissue disease	109 (0.8)	358 (0.3)	3.23 (2.91-3.59)
Systemic lupus erythematosus	27 (0.2)	94 (0.1)	2.35 (1.90-2.91)
Sjögrens syndrome	41 (0.3)	119 (0.1)	3.56 (2.99-4.24)
Systemic sclerosis	5 (< 0.1)	29 (< 0.1)	1.83 (1.21-2.77)
Immune myopathies	8 (0.1)	22 (< 0.1)	3.86 (2.61-5.70)
Vasculitis	34 (0.2)	113 (0.1)	3.87 (3.19-4.68)
Inflammatory bowel disease	202 (1.4)	1,094 (0.9)	1.62 (1.50-1.74)
Celiac disease	57 (0.4)	181 (0.2)	2.96 (2.54-3.44)
Immune-mediated thyroiditis	57 (0.4)	326 (0.3)	1.36 (1.18-1.56)
Hashimoto thyroiditis	21 (0.1)	70 (0.1)	2.20 (1.71-2.83)
Graves disease	37 (0.3)	260 (0.2)	1.14 (0.97-1.35)
Immune-mediated CNS disorders or neuropathies	52 (0.4)	361 (0.3)	1.39 (1.22-1.59)
Guillain-Barré syndrome	7 (< 0.1)	44 (0.0)	1.79 (1.25-2.56)
Multiple sclerosis	45 (0.3)	279 (0.2)	1.56 (1.35-1.80)
Myasthenia gravis	< 5	39 (< 0.1)	Not estimable
Diabetes mellitus type 1	323 (2.3)	1,439 (1.2)	1.97 (1.85-2.09)
Addison’s disease	< 5	16 (< 0.1)	Not estimable
Psoriasis	197 (1.4)	1,086 (0.9)	1.54 (1.43-1.66)
Immune-mediated cytopenia	38 (0.3)	79 (0.1)	4.59 (3.76-5.61)

Data are presented as No. or No. (%) unless otherwise indicated. IMD = immune-mediated disease; NA = not applicable.

a Defined as at least two International Classification of Diseases-coded inpatient or outpatient visits in the National Patient Register before sarcoidosis diagnosis.

### Familial Aggregation

**Figure 1:**
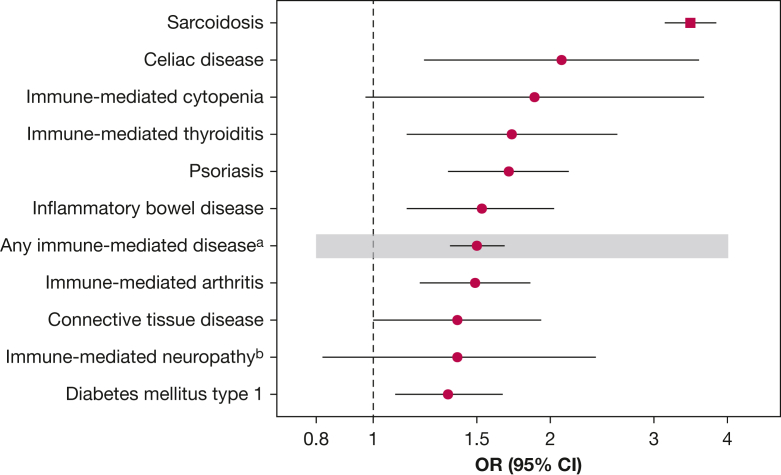
Graph showing ORs and 95% CIs of sarcoidosis in probands associated with sarcoidosis or immune-mediated disease in first-degree relatives. ^a^Includes all presented diseases except for sarcoidosis. ^b^Includes immune-mediated CNS disorders and neuropathies.

As shown in Table 4 and Figure 1, sarcoidosis was the most common of diseases ascertained in FDRs and had the highest familial OR (OR, 3.46; 95% CI, 3.13-3.83). Having an FDR with a diagnosis of any IMD (excluding sarcoidosis) was associated with 50% higher odds of developing sarcoidosis (OR, 1.50; 95% CI, 1.35-1.67) (Table 4, Fig 1). This was true for all IMDs or IMD groups. Celiac disease in FDRs was associated strongly with sarcoidosis in the probands (OR, 2.09; 95% CI, 1.22-3.58). Immune-mediated cytopenia was associated with 1.9-times higher odds of sarcoidosis, followed by 1.7-fold increased odds for thyroiditis and skin psoriasis. Higher odds of sarcoidosis of about 50% were observed in probands with FDRs with a diagnosis of an inflammatory bowel disease (OR, 1.53; 95% CI, 1.14-2.03) and immune-mediated arthritis (OR, 1.49; 95% CI, 1.20-1.85). Familial ORs also were higher for immune-mediated CNS disorders or neuropathies (1.39; 95% CI, 0.82-2.39), connective tissue disease (1.39; 95% CI, 1.00-1.93), and diabetes mellitus type 1 (1.34; 95% CI, 1.09-1.66) (Table 4, Fig 1).

**Table 4:** ORs of Sarcoidosis in the Proband Associated With Immune-Mediated Disease in FDRs

Disease in FDRs	No. of FDRs With Disease (% of FDRs)		OR (95% CI)
	Probands With Sarcoidosis	Control Group Probands	
No. of FDRs	73,860	618,106	NA
Sarcoidosis	553 (0.75)	1,343 (0.22)	3.46 (3.13-3.83)
Any immune-mediated disease^a^	354 (0.48)	1,983 (0.32)	1.50 (1.35-1.67)
Immune-mediated arthritis	89 (0.12)	509 (0.08)	1.49 (1.20-1.85)
Connective tissue disease	38 (0.05)	227 (0.04)	1.39 (1.00-1.93)
Inflammatory bowel disease	47 (0.06)	248 (0.04)	1.53 (1.14-2.03)
Celiac disease	15 (0.02)	57 (0.01)	2.09 (1.22-3.58)
Immune-mediated thyroiditis	23 (0.03)	118 (0.02)	1.72 (1.14-2.60)
Immune-mediated CNS disorders or neuropathies	14 (0.02)	84 (0.01)	1.39 (0.82-2.39)
Diabetes mellitus type 1	92 (0.12)	575 (0.09)	1.34 (1.09-1.66)
Psoriasis	80 (0.11)	397 (0.06)	1.70 (1.34-2.15)
Immune-mediated cytopenia	9 (0.01)	39 (0.01)	1.88 (0.97-3.65)

FDR = first degree relative; NA = not applicable.

a Excluding sarcoidosis.

### Kinship, Sex, and Treatment Status

Having a full sibling with sarcoidosis was associated with a higher OR of sarcoidosis in the proband compared with having parents or offspring with the disease (Table 5). The same was true for diabetes mellitus type 1, but no differences were observed for other IMDs based on kinship. No differences in ORs were found either when we considered separately male and female proband patients and control participants (Table 5). In addition, IMDs in FDRs conferred the same ORs of sarcoidosis that was treated and not treated around diagnosis, except for psoriasis, for which the familial odds were 2.7-fold higher for treated sarcoidosis (OR, 2.67; 95% CI, 1.71-4.17) compared with being 1.4-fold higher for sarcoidosis that was not treated around diagnosis (OR, 1.41; 95% CI, 0.91-2.17) (Table 6).

**Table 5:** ORs of Sarcoidosis in the Proband Associated With Immune-Mediated Disease in FDRs by Kinship and Sex of the Proband

Disease in FDRs	Kinship				Sex		
	OR (95% CI)			*P* for Interaction	OR (95% CI)		*P* for Interaction
	Parent	Full Sibling	Child		Female Proband	Male Proband	
Sarcoidosis	3.29 (2.77-3.91)	4.25 (3.64-4.96)	2.21 (1.74-2.81)	< .001	3.60 (3.10-4.18)	3.36 (2.93-3.84)	.50
Any immune-mediated disease^a^	1.47 (1.25-1.72)	1.54 (1.31-1.81)	1.47 (1.09-1.98)	.91	1.59 (1.36-1.86)	1.43 (1.23-1.65)	.33
Immune-mediated arthritis	1.73 (1.28-2.35)	1.13 (0.79-1.63)	1.78 (0.98-3.24)	.18	1.51 (1.09-2.09)	1.48 (1.11-1.97)	.93
Connective tissue disease	1.51 (0.98-2.33)	1.34 (0.78-2.30)	0.95 (0.28-3.20)	.77	1.25 (0.74-2.11)	1.49 (0.98-2.27)	.60
Inflammatory bowel disease	1.43 (0.86-2.38)	1.84 (1.24-2.73)	1.13 (0.57-2.22)	.43	1.90 (1.30-2.78)	1.22 (0.79-1.88)	.13
Celiac disease	1.43 (0.51-4.04)	2.79 (1.22-6.35)	1.85 (0.63-5.44)	.60	2.37 (1.10-5.12)	1.81 (0.83-3.91)	.63
Immune-mediated thyroiditis	1.70 (0.79-3.64)	1.92 (1.14-3.24)	1.03 (0.26-4.11)	.71	1.86 (0.96-3.59)	1.64 (0.97-2.80)	.78
Immune-mediated CNS disorders or neuropathies	2.46 (1.11-5.48)	0.95 (0.41-2.21)	0.64 (0.09-4.67)	.19	1.91 (0.99-3.69)	0.94 (0.38-2.34)	.22
Diabetes mellitus type 1	1.04 (0.77-1.39)	2.25 (1.63-3.09)	1.21 (0.58-2.51)	.002	1.43 (1.05-1.95)	1.28 (0.96-1.70)	.60
Psoriasis	1.85 (1.28-2.67)	1.33 (0.93-1.90)	2.49 (1.40-4.45)	.13	1.79 (1.27-2.53)	1.63 (1.18-2.24)	.70
Immune-mediated cytopenia	Not estimable	Not estimable	Not estimable	Not estimable	Not estimable	Not estimable	Not estimable

FDR = first degree relative.

a Excluding sarcoidosis, but including immune-mediated cytopenia.

**Table 6:** ORs of Treated and Untreated Sarcoidosis in the Proband Associated With Immune-Mediated Disease in FDRs

Disease in FDRs	OR (95% CI)		*P* for Interaction
	Treated Sarcoidosis	Untreated Sarcoidosis	
Sarcoidosis	3.08 (2.44-3.89)	3.86 (3.22-4.63)	.13
Any immune-mediated disease^a^	1.92 (1.55-2.38)	1.30 (1.06-1.58)	.01
Immune-mediated arthritis	1.83 (1.20-2.80)	1.28 (0.86-1.91)	.23
Connective tissue disease	2.01 (1.08-3.74)	1.48 (0.83-2.63)	.48
Inflammatory bowel disease	1.76 (0.94-3.29)	1.42 (0.84-2.39)	.60
Celiac disease	3.42 (1.28-9.14)	1.44 (0.44-4.77)	.27
Immune-mediated thyroiditis	2.96 (1.31-6.67)	1.41 (0.66-3.02)	.19
Immune-mediated CNS disorders or neuropathies	2.21 (0.96-5.07)	0.54 (0.12-2.37)	.10
Diabetes mellitus type 1	1.27 (0.80-1.99)	1.09 (0.73-1.61)	.62
Psoriasis	2.67 (1.71-4.17)	1.41 (0.91-2.17)	.04
Immune-mediated cytopenia	Not estimable	Not estimable	Not estimable

FDR = first degree relative.

a Excluding sarcoidosis, but including immune-mediated cytopenia.

## Discussion

In this large register-based study, we observed that individuals with sarcoidosis are more likely to have a history of an IMD compared with individuals without sarcoidosis. As in the general population, IMDs such as diabetes mellitus type 1, immune-mediated arthritis, inflammatory bowel disease, and psoriasis were among the most common. Compared with control participants without sarcoidosis, however, IMDs like celiac disease and connective tissue disorders were more than three times more prevalent in the population with sarcoidosis, suggesting a strong association between these IMDs and sarcoidosis.

Our finding of higher odds of sarcoidosis associated with a history of a range of IMDs are in line with several studies in other populations (eg, from Taiwan,^4^ the United Kingdom,^20^ and the United States^21^), in which associations between sarcoidosis and celiac disease,^20^^,^^22^ inflammatory bowel diseases,^5^^,^^23^ ankylosing spondylitis,^24^ and psoriasis were reported.^25^ Evidence of these associations was reviewed previously.^3^ The mechanisms behind the co-occurrence of these diseases in an individual remain unclear. IMD treatment with biologic agents may trigger sarcoid-like reactions that may be mistaken for sarcoidosis.^26^ Other more likely factors are genetic predisposition (which is discussed herein) and dysregulation of the immune system. Evidence of such dysregulation has been reported in similar fashion in both sarcoidosis and other IMDs and may implicate one or several components of the immune system, such as regulatory T cells and natural killer cells.^27^, ^28^, ^29^, ^30^ In addition, dysbiosis of the gut microbiota may contribute to the onset of several IMDs,^31^ and preliminary findings suggest similar mechanisms in sarcoidosis whereby dysbiosis exerts influence on inflammatory cells through proinflammatory cytokines, leading to granuloma formation.^32^ All these mechanisms, especially the role of gut microbiota in IMD and sarcoidosis, need further exploration.

Our data also suggest familial aggregation of IMD signaling common genetic or environmental factor(s), or both, among sarcoidosis and IMDs. As a benchmark, sarcoidosis itself exhibited the highest familial OR, with an FDR having more than a threefold increased odds of sarcoidosis developing, as we previously showed using data covering the period up to 2013.^18^ Of all IMDs examined here, celiac disease in FDRs showed the strongest association with sarcoidosis in probands. Several previous studies using register-derived data from Sweden and one from Denmark showed similar results. Although data in most previous studies were limited to hospitalizations for sarcoidosis and other IMDs capturing only about 10% to 20% of all patients,^16^ the authors estimated higher risks of sarcoidosis in children of individuals with a diagnosis of rheumatoid arthritis,^10^ inflammatory bowel disease,^11^ diabetes mellitus type 1,^8^ and in FDRs of individuals with celiac disease,^13^ Graves disease,^9^ and multiple sclerosis.^12^^,^^33^

Data on the familial aggregation of sarcoidosis and IMDs in non-Scandinavian populations are scarce. Sarcoidosis and other IMDs likely coaggregate in other populations, but the strength of associations may be influenced by the prevalence of these diseases, the genetic composition of various populations, and the environmental forces behind different diseases. In the Swedish population, about 30% of patients with sarcoidosis are diagnosed with Löfgren syndrome, an acute but self-limiting form of sarcoidosis with a distinct genetic underpinning.^34^ We could not distinguish between patients with Löfgren syndrome and those without Löfgren syndrome using register data, and therefore ORs may not be readily transferable to populations in which proportions of patients with Löfgren sarcoidosis and those without Löfgren sarcoidosis differ considerably from ours.

A shared genetic predisposition for sarcoidosis and other IMDs is widely thought to explain at least some of the familial aggregation potential of these diseases. Indeed, human leukocyte antigen alleles are implicated strongly in the pathophysiologic characteristics of several IMDs (eg, in celiac disease^35^) and that of sarcoidosis.^34^^,^^36^ However, research is still very limited, and thus cannot allow us to understand fully the genetic architecture of sarcoidosis and that of other IMDs and allow for identification of similarities among diseases. Both sarcoidosis and most IMDs are multifactorial, and no single genetic variant or variants have been shown to explain the predisposition to these diseases. Some of the shared risk for sarcoidosis and IMDs in families likely is conferred by environmental factors, or a complex interplay between genetic and environmental (epigenetic) factors. Many environmental factors shared within families such as infections or lifestyle factors like dietary habits and smoking have been implicated in the cause of autoimmunity and that of IMDs.^37^ Finally, occupational exposures are known risk factors for both sarcoidosis^38^ and IMDs^39^ and tend to cluster in families, and their role warrants further investigation.

This study has some limitations worth mentioning. Despite the use of large registers with nationwide coverage, small numbers of some IMDs were found, which in some cases led to some imprecise estimates, or they did not allow us to examine rarer IMDs. In addition, we did not have data on diagnoses set in primary care, and we have likely missed cases of celiac disease, autoimmune thyroid disease, and skin psoriasis, thus underestimating their prevalence in patients with sarcoidosis and control participants.

Although registers provided a unique opportunity to identify relatively unbiased estimates of the history and familial coaggregation of IMDs in sarcoidosis, we did not have access to participants’ medical records to validate these diagnoses based on accepted diagnostic criteria. Therefore, some misclassification resulting from misdiagnosis of both sarcoidosis and that of IMDs or missing some individuals with these diseases is to be expected, as indicated herein. Misdiagnosis is expected to be more common for diseases that exhibited similar clinical presentation and nevertheless is unlikely, for example, in the case of celiac disease. If any, this type of misclassification has diluted some of the familial associations among IMDs and sarcoidosis. To mitigate the risk of this bias in our study, we included data from specialized outpatient clinics in addition to hospitalization data and restricted the study to individuals with at least two visits to the hospital or clinic listing an ICD code for the disease of interest. In this study, we focused on history of IMDs in patients with sarcoidosis, which may be of interest to physicians at diagnosis, but IMDs can occur even after sarcoidosis diagnosis, leading to higher risks of co-occurrence of sarcoidosis and IMDs.

## Interpretation

This large population-based study showed that IMDs are more common in individuals with sarcoidosis and aggregate in families with sarcoidosis, suggesting that there may be shared genetic and environmental factors that trigger pathways of autoimmunity. Further research should advance the knowledge of these factors and mechanisms, which we hope will lead to more effective diagnostic and treatment strategies for individuals affected by these challenging conditions.

## Funding/Support

This study was funded by the 10.13039/501100003793Swedish Heart-Lung Foundation (Hjärt-Lungfonden; project nos. 2017-0412 and 2020-0452). Sarcoidosis research is also supported by the Swedish Heart-Lung Foundation (project nos. 2022-0140 and 2022-0194), the 10.13039/501100004359Swedish Research Council (Vetenskapsrådet; project nos. 2017-01548, 2022-00826, and 2022-00870), the Sven and Ebba Hagberg Prize, Karolinska Institutet’s Research Foundation and Funds, and a regional agreement on medical training and clinical research (ALF) between Region Stockholm and Karolinska Institutet.

## Financial/Nonfinancial Disclosures

None declared.
